# Analysis of clozapine-induced seizures using the Japanese Adverse Drug Event Report database

**DOI:** 10.1371/journal.pone.0287122

**Published:** 2023-06-12

**Authors:** Masakazu Hatano, Kaho Yamada, Haruna Matsuzaki, Rina Yokoi, Takeo Saito, Shigeki Yamada

**Affiliations:** 1 Department of Pharmacotherapeutics and informatics, Fujita Health University School of Medicine, Toyoake, Aichi, Japan; 2 Department of Psychiatry, Fujita Health University School of Medicine, Toyoake, Aichi, Japan; Dokkyo Medical University School of Medicine, JAPAN

## Abstract

Among antipsychotics, clozapine is associated with a high risk of seizures. This study aimed to generate novel hypotheses regarding trends in the onset of clozapine-induced seizures using the JADER (Japanese Adverse Drug Event Report) database. Seizures were defined according to the Standardized MedDRA Queries (SMQ) for convulsions (SMQ20000079). Trends in the onset of clozapine-induced seizures were assessed using multivariate logistic regression analysis with covariates of sex, age, clozapine dose, antipsychotic polypharmacy, concomitant medications, and history of convulsive disorder. In addition, we assessed the time-to-onset of clozapine-induced seizures using the median time, interquartile range, and Weibull shape parameter. The JADER database registered 2,745 cases of adverse events with clozapine, and 1,784 cases were included in the analysis after excluding cases for which clinical information was not available. Medium (200–400 mg) and high (> 400 mg) doses of clozapine had a significantly higher reporting rate of seizures than low doses (< 200 mg) (adjusted reporting odds ratio [aROR] = 3.05, 95% confidence interval [CI]: 1.86–4.99 and aROR = 9.81, 95% CI: 6.06–15.89, respectively). Younger age, antipsychotic polypharmacy, and concomitant use of lithium were also significantly associated with reports of seizures. The time-to-onset analysis of 222 cases of clozapine-induced seizures showed that the median time was 134 (interquartile range, 72–295) days. The 95% CI of the WSP β-value for clozapine-induced seizures included 1 and was classified as a random failure type. In conclusion, the results suggest that clozapine-induced seizures are dose-dependent adverse events that should be monitored with consideration of the effects of age and concomitant medications. Further epidemiological research is needed to strengthen and validate our hypotheses.

## Introduction

Antipsychotics are the most useful treatment for schizophrenia. However, it is estimated that approximately 30% of patients with schizophrenia have treatment-resistant schizophrenia (TRS), which does not respond to antipsychotics [1]. Clozapine has superior efficacy compared to other antipsychotics [2] and is recommended as a therapeutic option for TRS in guidelines [3–5].

In terms of safety, clozapine has a unique adverse event profile among antipsychotics. In the clinical setting, central nervous system (CNS) abnormalities are often a problem with clozapine treatment. Electroencephalogram (EEG) abnormalities have been reported in 50–60% of cases [6–8], and the incidence of seizures ranges from 1–5% [8–10]. The risk has been shown to increase in a dose-dependent manner [9], strictly in a plasma concentration-dependent manner [11, 12]. Patients with a history of convulsive disorder have also been reported to be more likely to have seizures, even with low doses of clozapine [10]. In addition, other risk factors for CNS abnormalities include concomitant use of lithium and shorter illness duration before clozapine initiation [13]. EEG abnormalities do not necessarily cause seizures [14], and other factors may be associated with the incidence of clozapine-induced seizures. In contrast, since the frequency of seizures is not very high, it is difficult to analyze the associated factors because they are not sufficiently reported in general clinical trials with a limited number of patients. The use of clozapine is significantly restricted, prescription rates are extremely low, and case-control studies are insufficient to collect relevant cases.

A large sample size is needed to elucidate the characterization of rare adverse drug reactions (ADRs); therefore, we focused on the Japanese Adverse Drug Event Report (JADER) database. The JADER database is a spontaneous reporting system (SRS) for adverse events published by the Pharmaceuticals and Medical Devices Agency (PMDA) in Japan. The information sources are mainly spontaneous case reports from companies and medical institutions as well as serious ADRs in study reports derived from post-marketing clinical trials, drug-use-result surveys, and specified drug-use surveys [15]. The SRS can be useful to generate hypotheses about the detection of unknown or rare ADRs and their trends in onset by analyzing vast amounts of ADR data. Although there have been several studies for the association of clozapine with CNS abnormalities in the broad sense, little is known about the more severe ADRs, seizures, other than that they are plasma concentration-dependent and are predicted by a past history of convulsive disorder. Furthermore, the effects of concomitant use with various medications must be considered in clinical settings. To generate novel hypotheses for our clinical question, we analyzed medical big data regarding trends in the onset of clozapine-induced seizures.

## Materials and methods

### Data source

This pharmacovigilance study used the JADER database provided by the PMDA. We downloaded the JADER database, which was registered between April 2004 and November 2021, from the PMDA website [16]. The JADER database consists of four data tables: 1) patient demographic information (DEMO), 2) drug information (DRUG), 3) adverse event information (REAC), and 4) medical history (HIST). We built a relational database according to the patient identification number using R 4.2.0 (The R Foundation for Statistical Computing). The DRUG table is classified into three categories according to the relevance of the adverse event: “suspected drug,” “concomitant drug,” and “interaction.” We included all categories in our analysis.

Adverse events were coded using the preferred term (PT) by the Medical Dictionary for Regulatory Activities/Japanese version (MedDRA/J) version 25.0 [17]. Seizures was defined according to the narrow scope of the Standardized MedDRA Queries (SMQ) for convulsions (SMQ20000079 containing 101 PTs) (S1 Table).

Ethics approval and consent to participate were not required since this study was performed using an open access database.

### Analysis

#### Onset profile in clozapine-induced seizures

We analyzed the differences in clinical factors associated with seizures and other adverse events in clozapine cases to evaluate the onset profile of clozapine-induced seizures. We calculated the adjusted reporting odds ratio (aROR) and its 95% confidence interval (CI) using multivariate logistic regression analysis with the covariates of sex, age, clozapine dose, antipsychotic polypharmacy, concomitant medication (lithium, fluvoxamine, and antiepileptic agents), and history of convulsive disorder. A dataset of clozapine cases was used, and cases with unknown sex, age, clozapine dose, or lithium dose were excluded. Cases in which age was registered in a non-numeric format (e.g., child, adult, and elderly) were also excluded. Age was categorized into 10-year increments in the JADER dataset. We evaluated the age groups in the categories of 30–39, 40–49, 50–59, and 60 and older, with 29 and younger as the reference level. We categorized the clozapine dose as “low dose (< 200 mg)”, “medium dose (200–400 mg)”, and “high dose (> 400 mg)”, and used the low dose as the reference level. Antipsychotic polypharmacy was defined as the presence or absence of any of the other antipsychotics (approved by the Ministry of Health, Labor, and Welfare) combined with clozapine. Lithium and fluvoxamine were selected because they may increase the risk of seizures and clozapine plasma concentrations, respectively [13, 18]. We categorized the lithium dose as “no use”, “low dose (≦ 600 mg)”, and “high dose (> 600 mg)”, with no use as the reference level. Antiepileptic agents were defined as sodium valproate, carbamazepine, lamotrigine, clonazepam, or diazepam. Cases in which these antiepileptic medications were initiated after the onset of seizures were counted as nonconcomitant. We extracted cases in which convulsions (SMQ 20000079) were registered in the primary disease information in the HIST table and classified them as a history of convulsive disorder. The multivariate logistic regression model was as follows: case (seizures or other adverse events) ∼ sex (male or female) + age (29 and younger, 30–39, 40–49, 50–59, or 60 and older) + clozapine dose (low dose, medium dose, or high dose) + antipsychotic polypharmacy (yes or no) + lithium (no use, low dose, or high dose) + fluvoxamine (yes or no) + antiepileptic agents (yes or no) + history of convulsive disorder (yes or no). P-values were two-sided, and values < 0.05 were considered significant. All statistical analyses were performed using R 4.2.0 (The R Foundation for Statistical Computing).

#### Time-to-onset analysis

We assessed the time-to-onset of clozapine-induced seizures using the median time, interquartile range, and Weibull shape parameter (WSP). Time-to-onset data were calculated from the date of clozapine initiation and the date of seizure onset, and cases with missing values were excluded. The analysis period was 1,095 days, and cases in which clozapine-induced seizures occurred after that period were also excluded. The shape parameter β of the Weibull distribution represents a hazard without a reference population. The classification criteria were defined as follows: when the 95% CI of β included 1, the hazard was constant over time (random failure type); if the lower limit of the 95% CI of β was >1, the hazard increased over time (wear-failure type); if the upper limit of the 95% CI of β was <1, the hazard decreased over time (early failure type) [19]. Time-to-onset analysis was performed using JMP 13.0 (SAS Institute Inc., Cary, NC, USA).

## Results

**Fig 1:**
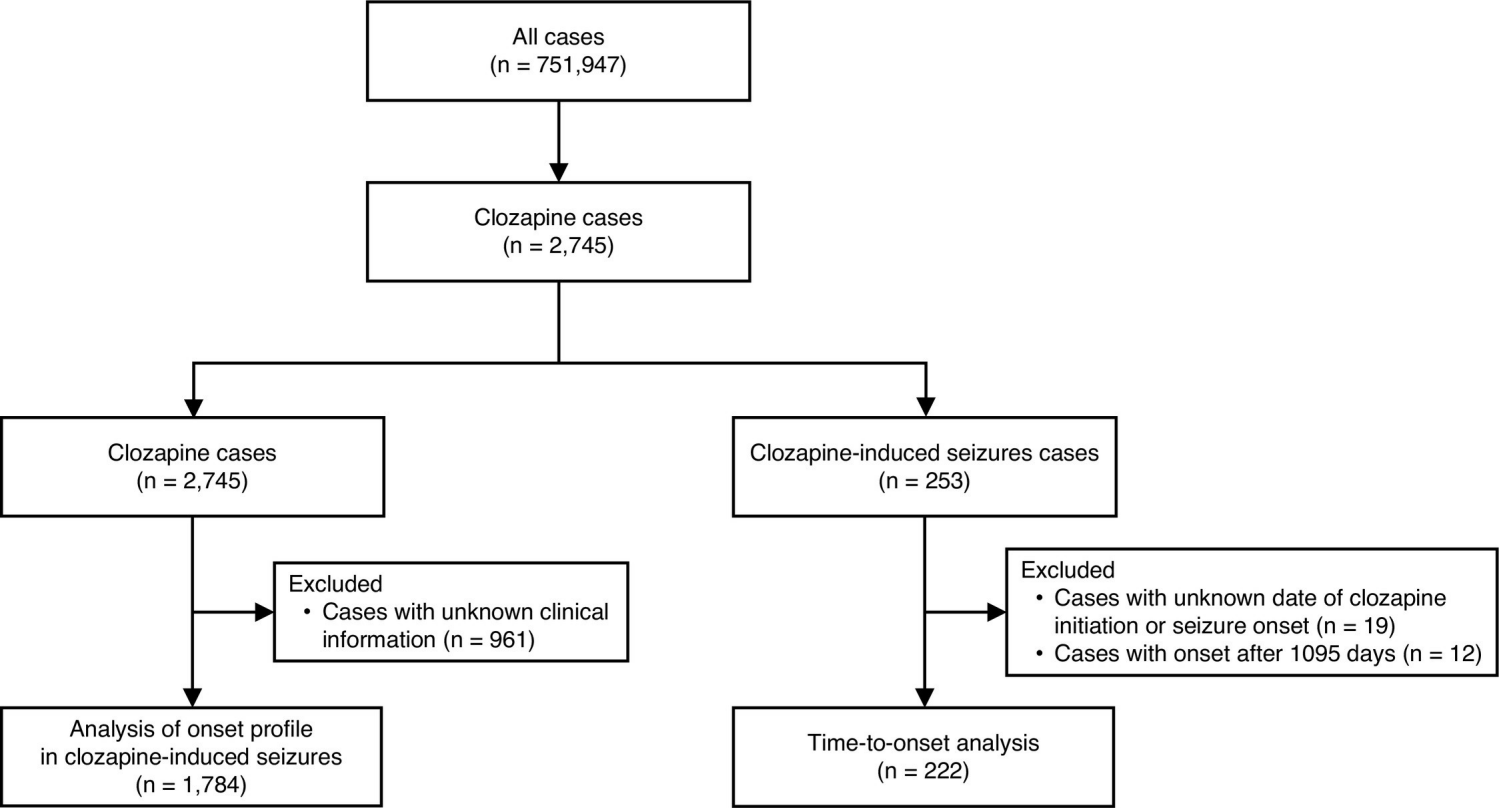
Flowchart of data analysis.

**Fig 2:**
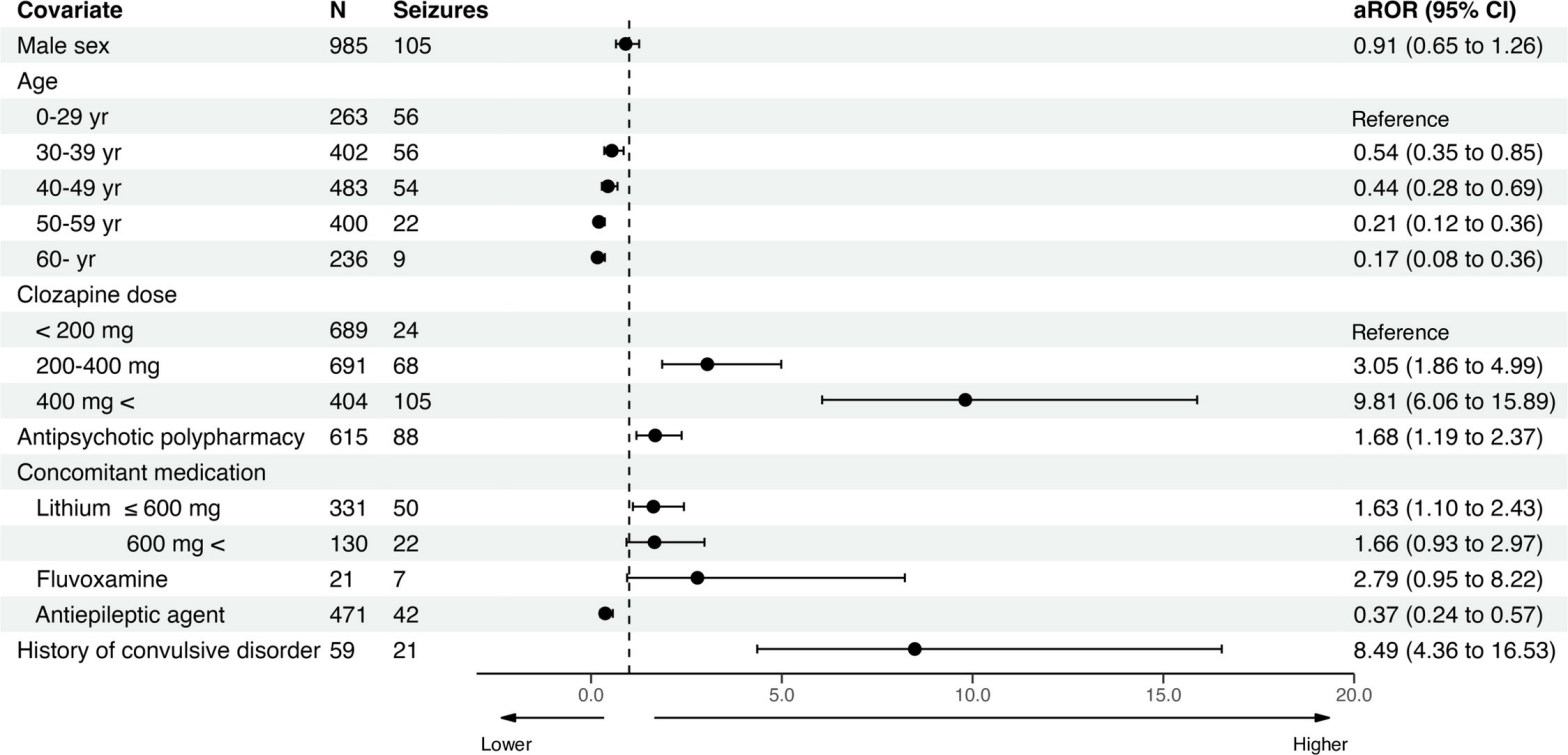
Onset profile in cases of clozapine-induced seizures using multivariate logistic regression analysis. aROR, adjusted reporting odds ratio; CI, confidence interval.

The data analysis process is illustrated in Fig 1. The JADER database registered 751,947 cases in the DEMO table between April 2004 and November 2021. The reported number of adverse events with clozapine use was 2,745 cases. After excluding cases in which sex, age, clozapine dose, or lithium dose were unknown, 1,784 cases were analyzed. In the data used in the multivariate logistic regression analysis, 197 cases of clozapine-induced seizures were included. All age groups 30 years and older had a significantly lower reporting rate of seizures than younger patients (< 30 years). Medium (200–400 mg) and high (> 400 mg) doses of clozapine had a significantly higher reporting rate of seizures than the low dose (< 200 mg) (aROR = 3.05, 95% CI: 1.86–4.99 and aROR = 9.81, 95% CI: 6.06–15.89, respectively). Antipsychotic polypharmacy had significantly high reporting rates of seizures (aROR = 1.68, 95% CI: 1.19–2.37). Compared to cases with no use of lithium, low dose (≦ 600 mg) had significantly high reporting rates of seizures (aROR = 1.63; 95% CI: 1.10–2.43), while high dose (> 600 mg) approached statistical significance (aROR = 1.66; 95% CI: 0.93–2.97; P = 0.086). Similarly, concomitant use of fluvoxamine approached statistical significance for association with a high reporting rate of seizures (aROR = 2.79; 95% CI: 0.95–8.22; P = 0.063). A history of convulsive disorder was found to be significantly associated with a high reporting rate of seizures (aROR = 8.49, 95% CI: 4.36–16.53). Conversely, the concomitant use of antiepileptic agents was associated with a significantly low reporting rate of seizures (aROR = 0.49, 95% CI: 0.33–0.72). The results of the multivariate logistic regression analysis are summarized in Fig 2.

**Fig 3:**
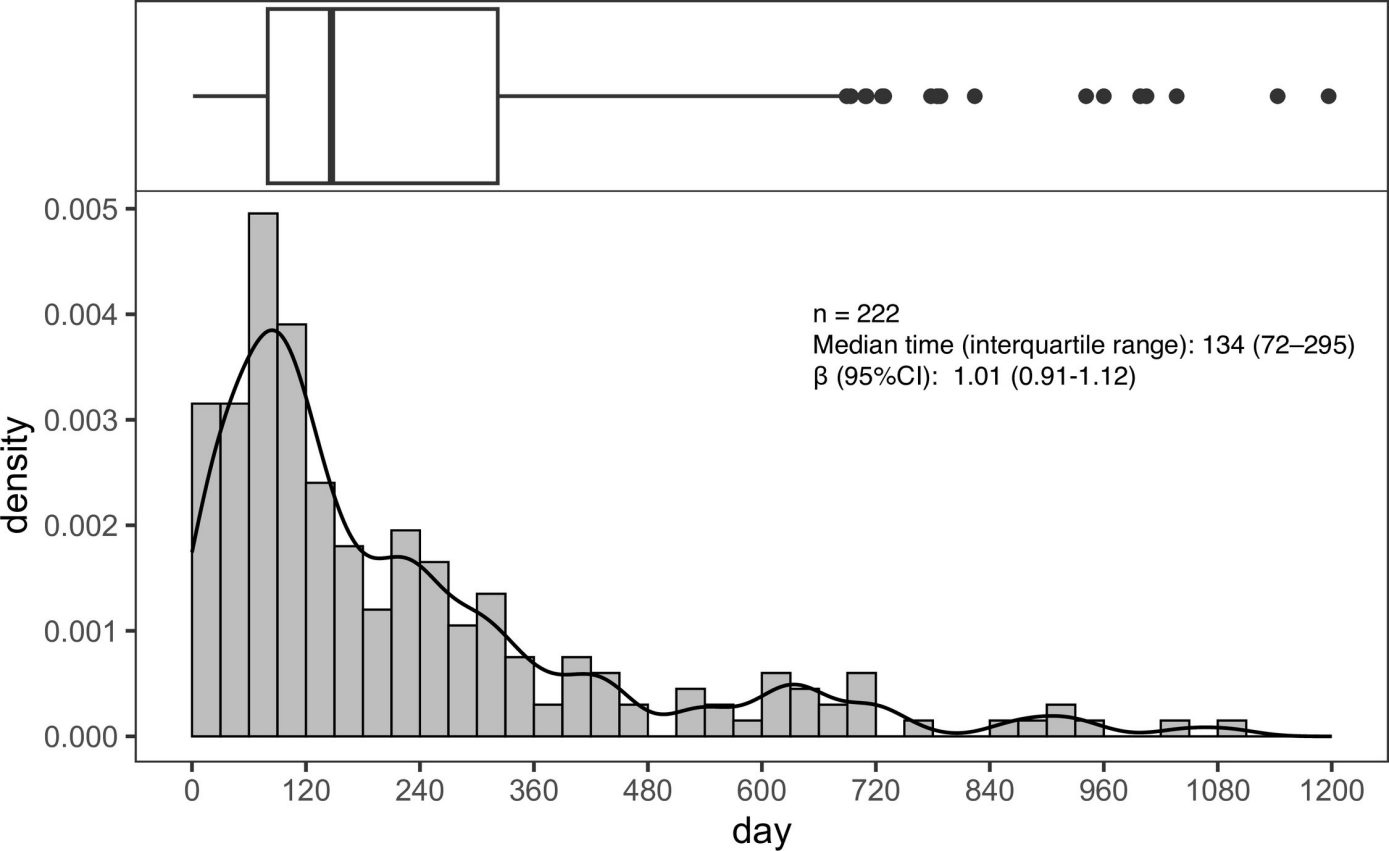
Histograms and Weibull shape parameters of clozapine-induced seizures. CI, confidence interval.

There were 253 cases of clozapine-induced seizures registered in JADER. The time-to-onset analysis was performed on 222 patients, excluding 19 cases in which the date of clozapine initiation or seizure onset was unknown and 12 cases in which seizures occurred after 1095 days. The median time for clozapine-induced seizures was 134 (interquartile range, 72–295) days. The observed rate of seizures within 1 year after the initiation of clozapine was 82.0% (182/222 cases). The 95% CI of the WSP β-value for clozapine-induced seizures included 1. A histogram (from day 0 to day 1095) and WSP analysis of clozapine-induced seizures are shown in Fig 3.

## Discussion

We evaluated trends in the onset of clozapine-induced seizures using the SRS for adverse events in Japan. We found a significant increase in the reporting rate of seizures in young patients and those with high-dose clozapine, antipsychotic polypharmacy, concomitant use of lithium, and a history of convulsive disorder. Fluvoxamine approached statistical significance for association with higher reporting rates of seizures. Clozapine-induced seizures are associated with higher plasma concentrations and have been shown to increase the risk of incidence at concentrations >1000 ng/mL [20]. Because plasma concentrations correlate with dose [21], the higher reporting rate of seizures with increasing doses was replicated herein. Similarly, our finding regarding a history of convulsive disorder supports previous studies [10].

Regarding other factors, evidence of an association with clozapine-induced seizures is limited to small-scale observational studies and case reports. Moreover, previous studies have examined the risk factors for CNS abnormalities, including EEG abnormalities, and few studies have focused on seizures alone. This may be because of the clinically low incidence of seizures, and thus, the lack of statistical power in the analysis of seizures alone. In a retrospective study, Kitagawa et al. reported that concomitant use of lithium is a risk factor for CNS abnormalities regardless of the plasma concentration of clozapine [13]. The mechanism underlying the interaction between lithium and clozapine is currently unknown. Since lithium has little effect on the pharmacokinetics of clozapine, this may be because lithium lowers the seizure threshold rather than increasing the plasma concentrations of clozapine. Our study also examined lithium dose but found little difference in aROR between low and high doses. Although these results suggest that lithium induces seizures regardless of the dose, further investigation is needed because of the small number of cases of high-dose concomitant use of lithium. Age has been shown to be associated with CNS abnormalities in two observational studies, with younger patients at a higher risk [22, 23]. However, CNS abnormalities have also been reported to be associated with the duration of illness in addition to age [13, 23]. Because of the generally high correlation between age and the duration of illness, the possibility of either being a confounding factor should be noted. To our knowledge, no studies have evaluated the effects of polypharmacy (clozapine plus other antipsychotics) on clozapine-induced seizures. A population-based cohort study in Korea evaluated the association between antipsychotic polypharmacy (not limited to clozapine) and seizures in pediatric patients [24]. Jeon et al.’s study showed that antipsychotic polypharmacy increases seizure risk, but the association disappeared in analyses adjusted for antipsychotic dosage, with inconsistent results. Our results suggest that polypharmacy may increase the risk of seizures, regardless of the clozapine dose. Although there is a concern that other concomitant antipsychotic doses were not considered, it is conceivable that polypharmacy could result in an additive increase in risk, since antipsychotics lower seizure thresholds in a small way [25]. We believe that the results of this study provide evidence to strengthen support for antipsychotic polypharmacy as a risk factor. Antipsychotic polypharmacy has been noted to increase the risk of various adverse events and thus should be avoided whenever possible in terms of seizures [26].

Conversely, concomitant use of antiepileptic medications was associated with a significantly lower rate of seizure reporting. Since the algorithm for clozapine treatment proposed by Schoretsanitis et al. states that prophylactic antiepileptic medication should be considered when clozapine plasma concentrations are > 600 ng/mL, it is clear that the concomitant use of antiepileptic drugs reduces the reporting rate of seizures [27]. In addition, sodium valproate decreases plasma concentrations of N-desmethylclozapine, a major metabolite of clozapine [28]. N-desmethylclozapine has been associated with dose-dependent adverse events, and its reduced plasma concentration may decrease the risk of seizures [29]. Carbamazepine also induces cytochrome P450 (CYP) 1A2 and 3A4, which may reduce the plasma concentrations of clozapine [30].

The median time to the onset of clozapine-induced seizures was 134 days. Clozapine treatment is regulated to require 18 weeks of hospitalization according to the Clozaril package insert in Japan, and clozapine is generally titrated from a low dose and increased to a maintenance dose by the time of discharge from the hospital. Therefore, it is possible that the seizure risk increased as the clozapine dose was increased, since the time to onset of seizures coincided with the time of the regulated discharge. Clozapine-induced seizures were classified as a random failure type in the WSP analysis, and seizure onset was also observed during the entire analysis period. These results suggest that even after clozapine is at the maintenance dose, further dose increases and concomitant use of lithium and other antipsychotics may be necessary, and continued careful monitoring is warranted.

This study has several limitations. First, the factors in the analyzed onset profiles may be clinically inadequate. For example, smoking and coffee consumption are assumed to be important factors involved in the onset of seizures because they affect clozapine plasma concentrations [31, 32], but this information is not registered in the JADER database. Additionally, drug-drug interactions with medications used to treat non-psychiatric disorders (e.g., omeprazole, erythromycin, and rifampicin, which induce or inhibit CYP isoenzymes) [33] could not be analyzed because few concomitant cases were registered in the study. Fluvoxamine was included in this analysis because it increases the clozapine plasma concentration [18], but it may have been a false negative because there were not enough cases of concomitant use. A common limitation of the SRS is the lack of denominator information because only adverse event cases are registered [34]. To clarify, the trend in the onset of clozapine-induced seizures is being compared to cases of adverse events caused by clozapine other than seizures. The comparison is not being made to clozapine cases with no adverse events. Therefore, it should be noted that the aROR in this study cannot directly indicate the magnitude of the risk. Furthermore, not all adverse event cases have been reported (i.e., under-reporting); conversely, an increase in the number of reports due to post-marketing surveillance may have affected the results.

In conclusion, our results provide a novel hypothesis that younger age, antipsychotic polypharmacy, and concomitant use of lithium are associated with clozapine-induced seizures, in addition to the previously established evidence for the relationship between clozapine dose and history of convulsive disorder; although no significant differences were found concerning lithium dose or concomitant use of fluvoxamine, this is a candidate for future validation. We also found that most cases occur within the first year of clozapine initiation; however, continued monitoring is required thereafter. Further epidemiological research is needed to strengthen and validate the novel hypotheses obtained in this study.

## Supporting information

S1 Table Preferred terms in SMQ20000079 (Convulsions). (PDF)
