# Supplementary material for: Analysis of clozapine-induced seizures using the Japanese Adverse Drug Event Report database
Source: PLoS One. 2023 Jun 12;18(6):e0287122. doi: 10.1371/journal.pone.0287122 (PMC10259781; doi:10.1371/journal.pone.0287122)
Supplement: S1 Table — (PDF) [file pone.0287122.s001.pdf]

S1 Table. Preferred Terms in SMQ20000079 (Convulsions)

PT code	PT name
10082398	1p36 deletion syndrome
10078971	2-Hydroxyglutaric aciduria
10052075	Acquired epileptic aphasia
10076948	Acute encephalitis with refractory, repetitive partial seizures
10056347	Alcoholic seizure
10083857	Alpers disease
10079140	Aspartate-glutamate-transporter deficiency
10003628	Atonic seizures
10056699	Atypical benign partial epilepsy
10003831	Automatism epileptic
10049612	Autonomic seizure
10054895	Baltic myoclonic epilepsy
10067866	Benign familial neonatal convulsions
10070530	Benign rolandic epilepsy
10071434	Biotinidase deficiency
10083005	CDKL5 deficiency disorder
10083749	CEC syndrome
10078827	CSWS syndrome
10075606	Change in seizure presentation
10053398	Clonic convulsion
10082716	Congenital bilateral perisylvian syndrome
10052391	Convulsion in childhood
10010920	Convulsions local
10010927	Convulsive threshold lowered
10012177	Deja vu
10073490	Double cortex syndrome
10013634	Dreamy state
10013752	Drug withdrawal convulsions
10071545	Early infantile epileptic encephalopathy with burst-suppression
10014129	Eclampsia
10015034	Epilepsia partialis continua
10015037	Epilepsy
10086114	Epilepsy of infancy with migrating focal seizures
10079824	Epilepsy surgery
10081179	Epilepsy with myoclonic-atonic seizures
10015049	Epileptic aura
10059232	Epileptic psychosis
10084187	Faciobrachial dystonic seizure
10016284	Febrile convulsion
10079438	Febrile infection-related epilepsy syndrome
10079424	Focal dyscognitive seizures
10049424	Frontal lobe epilepsy

S1 Table. Preferred Terms in SMQ20000079 (Convulsions) (cont)

PT code	PT name
10083933	GM2 gangliosidosis
10082918	Gelastic seizure
10083376	Generalised onset non-motor seizure
10018100	Generalised tonic-clonic seizure
10078727	Glucose transporter type 1 deficiency syndrome
10082084	Grey matter heterotopia
10085010	Hemiconvulsion-hemiplegia-epilepsy syndrome
10078100	Hemimegalencephaly
10071394	Hyperglycaemic seizure
10072456	Hypocalcaemic seizure
10048803	Hypoglycaemic seizure
10073183	Hyponatraemic seizure
10071081	Idiopathic generalised epilepsy
10021750	Infantile spasms
10084303	Jeavons syndrome
10085031	Juvenile absence epilepsy
10071082	Juvenile myoclonic epilepsy
10054030	Lafora's myoclonic epilepsy
10048816	Lennox-Gastaut syndrome
10076676	Migraine-triggered seizure
10069687	Molybdenum cofactor deficiency
10079825	Multiple subpial transection
10054859	Myoclonic epilepsy
10069825	Myoclonic epilepsy and ragged-red fibres
10082068	Neonatal epileptic seizure
10082067	Neonatal seizure
10085882	PURA syndrome
10085326	Parietal lobe epilepsy
10061334	Partial seizures
10056209	Partial seizures with secondary generalisation
10034759	Petit mal epilepsy
10086294	Photosensitive seizure
10073489	Polymicrogyria
10076982	Post stroke epilepsy
10076981	Post stroke seizure
10036312	Post-traumatic epilepsy
10052470	Postictal headache
10052469	Postictal paralysis
10070669	Postictal psychosis
10048727	Postictal state
10086607	Progressive encephalopathy, hypsarrhythmia and optic atrophy syndrome

S1 Table. Preferred Terms in SMQ20000079 (Convulsions) (cont)

PT code	PT name
10073487	Schizencephaly
10039906	Seizure
10039907	Seizure anoxic
10071350	Seizure cluster
10071048	Seizure like phenomena
10073677	Severe myoclonic epilepsy of infancy
10040703	Simple partial seizures
10086468	Sleep related hypermotor epilepsy
10041962	Status epilepticus
10063894	Sudden unexplained death in epilepsy
10043209	Temporal lobe epilepsy
10051171	Tonic clonic movements
10043994	Tonic convulsion
10075125	Tonic posturing
10073488	Topectomy
10081728	Transient epileptic amnesia
10080584	Tuberous sclerosis complex
10045476	Uncinate fits
